# North American boreal forests are a large carbon source due to wildfires from 1986 to 2016

**DOI:** 10.1038/s41598-021-87343-3

**Published:** 2021-04-08

**Authors:** Bailu Zhao, Qianlai Zhuang, Narasinha Shurpali, Kajar Köster, Frank Berninger, Jukka Pumpanen

**Affiliations:** 1 Department of Earth, Atmospheric, and Planetary Sciences, Purdue University, West Lafayette, IN 47907 USA; 2 Department of Agronomy, Purdue University, West Lafayette, IN 47907 USA; 3 Production Systems - Milk Production Unit, Natural Resources Institute Finland (Luke), Halolantie 31 A, Maaninka, FI-71750 Finland; 4 Department of Forest Sciences, University of Helsinki, PO Box 27, 00014 Helsinki, Finland; 5 Department of Environmental and Biological Sciences, University of Eastern Finland, PO Box 111, 80101 Joensuu, Finland; 6 Department of Environmental and Biological Sciences, University of Eastern Finland, PO Box 1627, 70211 Kuopio, Finland

**Keywords:** Boreal ecology, Fire ecology

## Abstract

Wildfires are a major disturbance to forest carbon (C) balance through both immediate combustion emissions and post-fire ecosystem dynamics. Here we used a process-based biogeochemistry model, the Terrestrial Ecosystem Model (TEM), to simulate C budget in Alaska and Canada during 1986–2016, as impacted by fire disturbances. We extracted the data of difference Normalized Burn Ratio (dNBR) for fires from Landsat TM/ETM imagery and estimated the proportion of vegetation and soil C combustion. We observed that the region was a C source of 2.74 Pg C during the 31-year period. The observed C loss, 57.1 Tg C year^−1^, was attributed to fire emissions, overwhelming the net ecosystem production (1.9 Tg C year^−1^) in the region. Our simulated direct emissions for Alaska and Canada are within the range of field measurements and other model estimates. As burn severity increased, combustion emission tended to switch from vegetation origin towards soil origin. When dNBR is below 300, fires increase soil temperature and decrease soil moisture and thus, enhance soil respiration. However, the post-fire soil respiration decreases for moderate or high burn severity. The proportion of post-fire soil emission in total emissions increased with burn severity. Net nitrogen mineralization gradually recovered after fire, enhancing net primary production. Net ecosystem production recovered fast under higher burn severities. The impact of fire disturbance on the C balance of northern ecosystems and the associated uncertainties can be better characterized with long-term, prior-, during- and post-disturbance data across the geospatial spectrum. Our findings suggest that the regional source of carbon to the atmosphere will persist if the observed forest wildfire occurrence and severity continues into the future.

## Introduction

Boreal forests are important in the global carbon (C) cycling since these ecosystems store one-third of the global terrestrial C^1^ and prevalent wildfires accelerate their C release into the atmosphere^2^. Massive amounts of C are released directly through biomass combustion. Post-fire C dynamics leading to increased heterotrophic respiration (R_H_) and decreased net primary production contribute to the C loss, shifting boreal forests from a C sink to a source^3^. Previous studies have shown that wildfires also significantly increased global land annual mean surface temperature in the twentieth century by 0.18 °C^4^. The warmer climate resulting from anthropogenic greenhouse gases and aerosol emissions has caused larger burned area in Canadian forests^5^. Within the last four decades, twice larger burned area and twice higher frequency of large fire events (> 1000 km^2^) in Canada have been reported^6^. These observational studies indicate that there is a positive feedback between wildfires and the global climate.

Wildfires influence the C dynamics in the boreal forests of North America (NA) partially through removing aboveground vegetation, since the regional forest plant species are susceptible to crown fires^7,8^. After severe fires, forests could temporarily shift to grasslands^9^. Alternatively, in response to the changes in temperature and moisture conditions as well as soil organic layer thickness, the newly-emerged dominant tree species might be different from the pre-fire community^10–12^. In either case, following the reduction of leaf area after the fire, the mass and energy fluxes between the biosphere and atmosphere will change, further influencing soil moisture, temperature and C dynamics^4,9,13^.

Wildfires also dramatically affect soil C storage and ecosystem C balance^14,15^. Soil organic matter combustion could release massive amounts of C to the atmosphere in severe fires. Nearly 90% of total combusted C in a North American boreal fire in 2014 was from soils (e.g., ref^16^). Together with immediate fire emissions from soils, the post-fire soil C emissions through soil respiration could further imbalance the C budget. The soil respiration is determined by soil thermal and moisture conditions and microbial community, which are all altered by fire^17^. Fires result in higher thermal conductivity in the ground and lower albedo by removing plant tissues and the organic layer on the surface^4,18,19^. This would increase soil temperature due to increasing solar radiation on the soil surface after the fire^13,20^. Soil water conditions after the fire will depend on the severity of fire because the density of trees and belowground vegetation determines the ecosystem evapotranspiration and the overland water flow^21^. For example, no soil moisture change was observed in a less severely burned forest in central Colorado in 2002, while a severely burned forest in this region had high soil moisture^20^. The shift in dominant microbial members, lower soil moisture and C storage collectively affect long-term post-fire CO_2_ emissions^22^. For example, soil CO_2_ efflux would initially reduce and then increase for several decades^23^, mainly due to the dynamics of soil C and fungi biomass recovery after the fire^24^.

Although the influence of fire on the boreal C budget has been previously modeled^2,25–27^, several limitations in these studies are evident. First, fire-induced CO_2_ emissions in many boreal regions, such as Russia^25,28^, Alaska^27^, Canada^29^ and the Northern Hemisphere as a whole^30^ have primarily focused on immediate combustion emission estimates. However, long-term post-fire soil emissions and NPP changes could account for a large proportion of total fire-related C loss^29^. Second, for both during- and post-fire C emissions, a few site-level studies are conducted based on field measurements^16,22,24^. At regional scales, process-based models are necessary when site-level observations are limited^26^. Third, although burn severity is an important control of C emissions, regional estimations are rare and records are limited^25^. Burn severity can be expressed as the fraction^31^ or amount^25^ of pre-fire ecosystem C lost during the fire. Unfortunately, burn severity information is not available in existing fire datasets (AICC, CWFIS, see SI and methods for details). When estimating regional C combustion, an average severity is generally assumed for an entire region^26,27,30^ or biome type^32^. These severity estimates are based on data published in the literature, limited available field data or expert knowledge, while the actual burn severity could differ dramatically among fires^33^.

To overcome these limitations mentioned above, we applied a process-based model, the Terrestrial Ecosystem Model (TEM;^34^), to understand the role of fire disturbance on the C budget of North American boreal forests using burn severity data retrieved from satellite images. Difference Normalized Burn Ratio (dNBR) from LANDSAT imagery was used to represent burn severity, which was used to estimate the proportion of vegetation and soil removal by fire. We have thus extracted burn severity information for all fires during 1986–2016. We conducted regional simulations for the study period and evaluated the spatial and temporal C dynamics considering fire impacts on C emissions, soil physics, soil nutrient status, and the subsequent net ecosystem production. Different from previous modelling studies, this study uses burn severity indices for all fires during the study period. We are interested in (a) the fire regime during the study period; (b) the way that fire impacts ecosystem C balance both spatially and temporally; (c) the influence of burn severity on C balance and on the emission patterns. We hypothesize that the during- and post-fire influences on the vegetation and soil and resultant C and N dynamics vary depending upon the burn severity.

## Results

### Fire regime during 1986–2016

**Figure 1:**
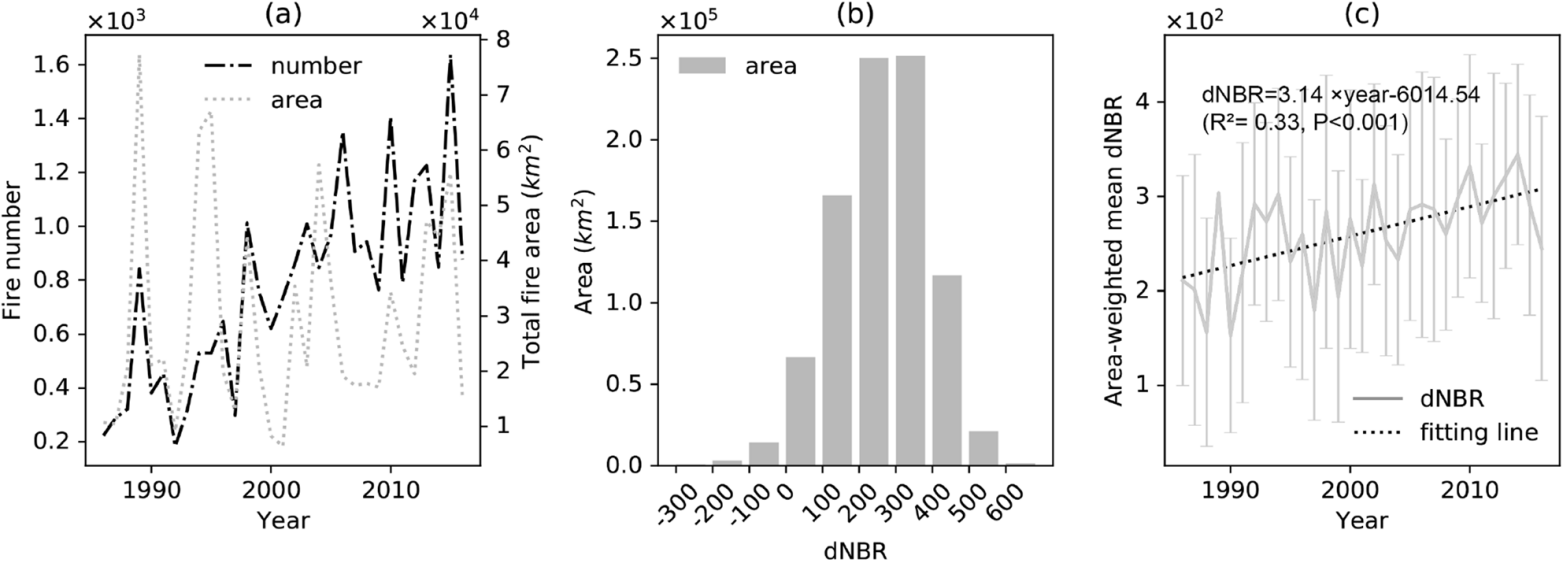
Summary of the fire regime during 1986–2016 in NA boreal forests: (**a**) variations of the fire area and fire number. (**b**) Histograms of dNBR (difference Normalized Burn Ratio), i.e., burn severity. The heights of grey bars are the total area of fires in which average dNBR is within the threshold indicated by the x axis. (**c**) Annual dNBR variation and trend of the fires. The grey line represents the mean while error bars represent the standard deviation. The mean values are linearly regressed to generate the fitting line.

Although the average fire interval in boreal forests is 80 years^35^, the areas burned more than once in the 31-year period of 1986–2016 still accounted for 4.8% of the total burned area (Supplementary Table S1). During this period, the number of fires generally increased, while the annual burned area didn’t show an increasing trend despite a large amplitude (the difference between the largest and smallest burned areas) (Fig. 1a). For most of the burned areas, the average dNBR value was 200–400, with an overall area-weighted average of 272.52 (Fig. 1b). Although the dNBR varied greatly within a year, annual area-weighted dNBR significantly increased during the 31-year period (Fig. 1c).

### Spatial patterns of fire impacts on ecosystem C balance

**Figure 2:**
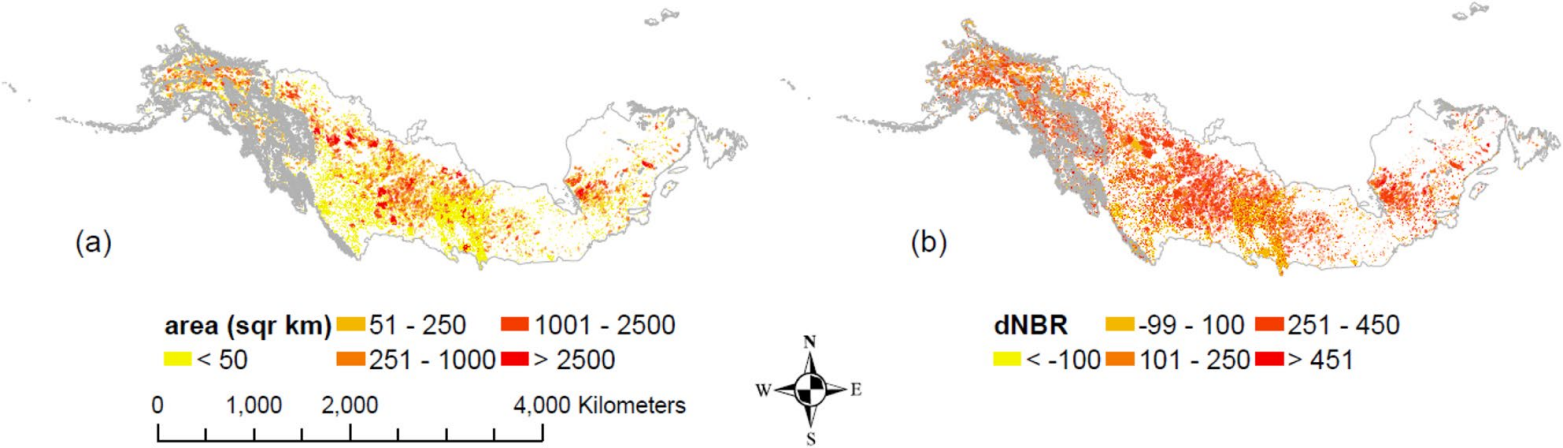
Fire area and burn severity (as dNBR) during 1986–2016 in NA boreal forest. (**a**) Fire area in km^2^. (**b**) Burn severity measured by the mean dNBR value within its perimeter. For both panels, the grey lines show the boundary of boreal forest. This figure was created by ArcMAP 10.7.1, (https://www.esri.com/en-us/arcgis/products/arcgis-maps-for-office/download).

**Figure 3:**
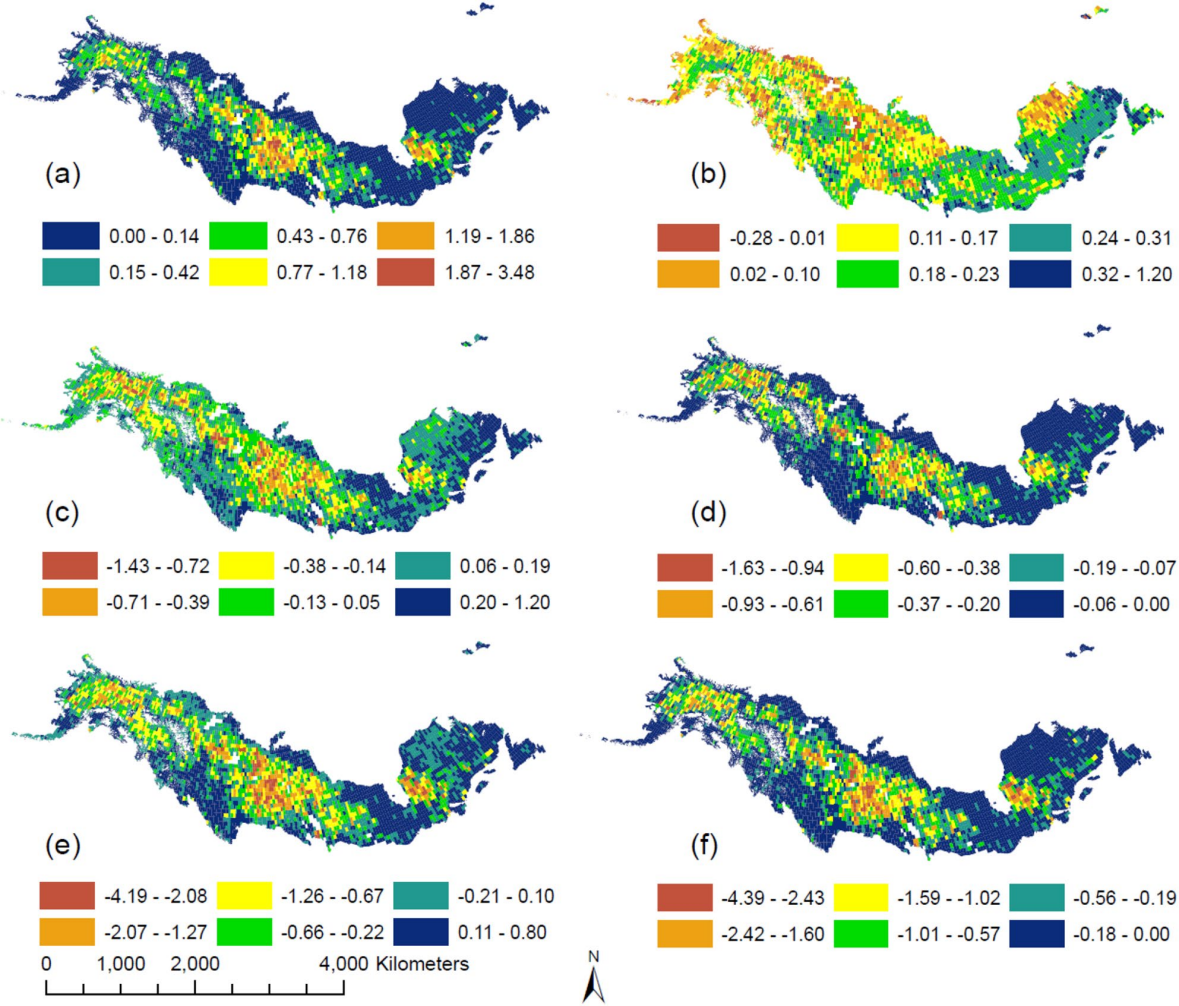
Spatial pattern of C sequestration and emission, accumulated for 1986–2016 (Gg km^−2^): (**a**) total emissions from direct combustion (both vegetation and soil); (**b**) cumulative NEP without considering fires, i.e., the distribution of ecosystem C storage when there is no fire; (**c**) cumulative NEP considering fires; (**d**) difference of cumulative NEP with and without considering fires (c minus b); (**e**) changes in ecosystem C storage considering fires; (**f**) difference of ecosystem C storage between simulations with and without fires (the former minus the latter). This figure was created by ArcMAP 10.7.1, (https://www.esri.com/en-us/arcgis/products/arcgis-maps-for-office/download).

The spatial pattern of C emissions during combustion followed that of the fire area and severity (Figs. 2, 3a). In particular, the total combustion emissions of the North American boreal forests during 1986–2016 were 1769.8 Tg C (Supplementary Table S2), with hotpots in Saskatchewan and Quebec, Canada. When no fire disturbance was considered, the majority of forests acted as C sinks, with a total 31-year cumulative NEP of 1030.0 Tg C. In addition, C sequestration in this region was higher in the east than in the west (Fig. 3b). The spatial pattern of cumulative NEP under fire also followed the fire distribution pattern, since fires removed vegetation and soil C and reduced NPP (Fig. 3c). Although fire greatly reduced the productivity of boreal forests, the 31-year regional cumulative NEP was still positive (59.0 Tg C). Meanwhile, the spatial pattern of the difference between fire and no-fire NEP had a similar spatial pattern to fire events (Fig. 3d). In addition, spatial patterns of total C stocks were the same as NEP (Fig. 3b), since it was the difference between NEP (59.0 Tg C) and combustion emissions (1769.8 Tg C). Therefore, although the NA boreal forests showed signs of recovery with a positive regional cumulative NEP during the study period, they acted as a C source (Fig. 3e). Due to massive fire emissions and reduced post-fire productivity, the total ecosystem C stocks were reduced by 2740.8 Tg C during the 31-year period compared with the estimate without fires. The pattern of differences between the C stocks with and without fires was highly consistent with that of the fire emission (Fig. 3f).

### Temporal pattern of fire impacts on ecosystem C balance

Compared with the number of wildfire occurrences, fire area was more consistent with the fire emission patterns (Supplementary Fig. S1a, Fig. 1a). When fires were not taken into account, the simulated regional forest biomass and soil organic C stocks increased from 1986 to 2016, while an opposite trend was found when fire impacts were taken into account (vegetation C: 557.0 Tg for no-fire vs. − 468.9 Tg for fire, soil organic C: 589.5 Tg for no fire vs. − 1125.4 Tg for fire, Supplementary Fig. S1b,c). Although the mean burn severity increased during the study period (Fig. 1c), the combustion emissions did not show such a trend due to a wide variation in the burned area. With and without fires, the estimated annual regional NPP, R_H_ and their differences, i.e., NEP, highly varied and were generally synchronous with each other (Supplementary Fig. S1e,f). When fires were taken into account in the simulation, NPP was always lower than that without fires, and their differences increased with year over the study period (Supplementary Fig. S1g). This was attributed to the removal of plant biomass due to fires. The difference in vegetation C storage (proportional to vegetation biomass) between the two scenarios grew larger with time (Supplementary Fig. S1b).

In contrast, R_H_ with fire regimes considered was generally higher before 2000, and similar in the early 2000s, suggesting that, despite the lower soil organic C storage with fires, other factors (e.g., soil temperature and moisture) might stimulate soil respiration. However, since the later 2000s, R_H_ decreased with fires because the reduced soil organic C overwhelmed the effect of soil temperature and moisture changes (Supplementary Fig. S1c,h).

The trend in NEP differences between fires and no-fires was more consistent with the difference in NPP than in R_H_ since NPP was larger in magnitude (Supplementary Fig. S1i). By 2016, fires resulted in a lower cumulative NEP by 971.0 Tg C than that under the no-fire scenario in the region.

### Influence of burn severity

**Figure 4:**
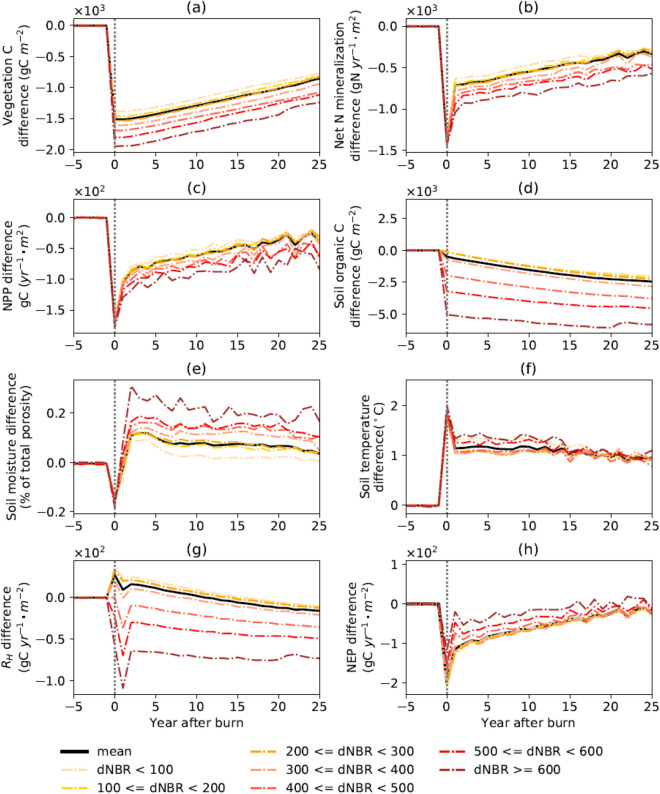
Difference of the changes in carbon pools and fluxes between the fire and the no-fire scenarios (the no-fire minus the fire scenario) under different levels of burn severity. Only cohorts burned once (95.2% of the total burned area) are used for calculation. The value of each curve is the average of all cohorts with corresponding dNBR values: (**a**) vegetation C (gC m^−2^); (**b**) annual net N mineralization (gN year^−1^ m^−2^); (**c**) annual NPP (gC year^−1^ m^−2^); (**d**) soil organic C (gC m^−2^); (**e**) soil moisture (% of total porosity); (**f**) soil temperature (°C); (**g**) Rh (gC year^−1^ m^−2^); (h) NEP (gC year^−1^ m^−2^).

According to the dNBR values and frequency (Fig. 1b), burn severity was classified into seven levels with an interval of 100 for comparison (Fig. 4). On average, wildfires removed 1512.0 g C m^−2^ of vegetation C in the region, with higher burn severity leading to higher removal rate (ranging between 1382.3 and 1951.4 g C m^−2^, Fig. 4a). Vegetation growth recovered steadily following fires, and by the 25th year, the difference in vegetation C between the fire and no-fire scenarios decreased to 773.0–1242.2 g C m^−2^. Net nitrogen (N) mineralization decreased on average by 1401.1 g N m^−2^ year^−1^ in the year of fire. Since the second year after the fire, the net N mineralization rate had increased and recovered by 1066.5 g N m^−2^ year^−1^ by the 25th year after fire (Fig. 4b). Similarly, the productivity of vegetation was reduced by 170.5 g C m^−2^ year^−1^ in the year of fire. However, after the fire, NPP increased regardless of burn severity with the subsequent vegetation regrowth. In the 25th year after the fire, the NPP difference between the two scenarios reduced by 132.6 g C m^−2^ year^−1^ (Fig. 4c).

Fire removed 499.1 g C m^−2^ of soil organic C. Compared with vegetation C, the removal of soil organic C showed more variations (ranging between 116.1 and 5057.1 g C m^−2^) as the severity class varied. However, unlike vegetation C, soil organic C decreased since the fire. The difference between the fire and the no-fire scenario increased to 2038.6–5827.1 g C m^−2^ in the 25th year after the fire. This was because the reduced vegetation provided less litter C to the soil so the soil organic C would reduce until vegetation fully recovers (Fig. 4d). Soil physical properties such as soil moisture and soil temperature in this research also changed after the fire. In particular, soil moisture (in % of total porosity) increased after fire, more under more severe fires. However, this change was small enough (ranging between − 0.07 and 0.08% in the first year after the fire while 0.005–0.17% in the 25th year after the fire) so that the soil moisture change had a trivial contribution to the dynamics of post-fire C budget (Fig. 4e). Soil temperature increased among all severity levels, with a range of 1.32–1.34 °C in the year after fire and 0.91–1.11 °C in the 25th year after the fire (Fig. 4f). In the first year after fire, R_H_ increased between 19.7 and 23.2 g C m^2^ year^−1^ for fires with the dNBR below 300, while decreased between 0.6 and 00109.2 g C m^2^ year^−1^ for fires with the dNBR above 300. However, even though the R_H_ for low-severity fires increased in the first few years after the fire, it decreased with time in response to the lower soil organic C. In particular, in the 25th year after the fire, the R_H_ increased between − 10.3 and − 13.0 g C m^2^ year^−1^ for fires with the dNBR below 300, and decreased between 20.9 and 73.1 g C m^2^ year^−1^ for fires with the dNBR above 300 (Fig. 4g).

NEP decreased after fire mainly due to less vegetation, ranging between 19.8 and 122.0 g C m^2^ year^−1^ with an average of 113.4 g C m^2^ year^−1^. The largest NEP decrease was found in when dNBR is below 100, where R_H_ increased most after the fire, while the smallest decrease was found in when dNBR is above 600, where R_H_ decreased the most. In the 25th year after the fire, with the increase of NPP and the decrease of R_H_, NEP increased and the difference between the fire and no-fire scenario decreased to − 19.8 to 8.8 g C m^2^ year^−1^ (Fig. 4h).

In addition, for all eight variables in Fig. 4 (vegetation C, net N mineralization, NPP, soil organic C, soil moisture, soil temperature, R_H_ and NEP), their averages (i.e., the black lines) were in close agreement with the two levels of burn severity of 100 ≤ dNBR < 200 and 200 ≤ dNBR < 300, since the majority of fires have severity within this range (Fig. 1b).

### Impact of burn severity on fire emission patterns

Under different burn severity levels, the primary sources of C emission were different. We further analyzed the proportion of vegetation and soil combustion during the fire, and the temporal pattern of post-fire emission (Supplementary Fig. S2a). When the burn severity was relatively low (dNBR < 300), the direct emission was dominated by vegetation combustion while the soil was almost unburned. Under more severe fires, soil combustion dominated the emission, which was 30.4 ± 14.8%, 56.1 ± 9.2%, 65.9 ± 6.8% and 72.0 ± 5.1% when dNBR was between 300–400, 400–500, 500–600 and above 600, respectively.

Since soils barely combusted when dNBR is below 300, the proportion of direct soil emission out of the total post-fire soil emission (i.e., direct soil emission plus accumulative R_H_ since fire) was close to 0 (Supplementary Fig. S2b). This value became larger with the increase in burn severity and the amount of soil combustion and declined after fire since the cumulative R_H_ accounted for a larger proportion. The contribution of direct soil emission out of soil total post-fire emission decreased from 78.0 to 13.9%, 92.1% to 25.6%, 93.1% to 49.9% and 97.9% to 67.7% when dNBR ranged between 300–400, 400–500, 500–600 and above 600, respectively. Notably, since severe fires tended to reduce post-fire R_H_, as we reported earlier, the proportion of direct soil emission decreased slower for severe fires. In particular, the value dropped by 64.1% (dNBR: 300–400), 66.5% (dNBR: 400–500), 43.2% (dNBR: 500–600), and 30.2% (dNBR: > 600) respectively, in the 25 years since burned.

The pattern of C emissions was similar among three relative low severity classes (dNBR < 300) and was different among the other four relative higher severity classes (Supplementary Fig. S2c) fires. Direct emissions accounted for a large portion of the total ecosystem emissions at the early stage after a fire with dNBR below 300 (87.3–89.0% at the year of fire), due to the combustion of vegetation. However, this proportion decreased quickly after the fire since R_H_ was hardly influenced and it made large contributions to the emission (37.4–39.6%). The pattern under severe burn was generally consistent between Supplementary Fig. S2b,c, as the emission from soil combustion became larger.

The difference between total emissions with and without considering fires and the direct emission is presented in this study as a ratio (Supplementary Fig. S2d). When the ratio is larger than one, a fire results in a higher proportion of indirect emissions via R_H_. However, when the ratio is close to one, the fire even triggered a destruction of the standing vegetation and reduced post-fire R_H_. The lower severity corresponded with higher ratios in the early post-fire stage. With time, the ratio gradually dropped to 0 as the ecosystem recovered back to the pre-fire stage, unless the forest stand was replaced by the other vegetation types. However, for all severity classes, the ratio increased in the 25 years after the fire, suggesting that the ecosystem and vegetation were yet to recover to the pre-fire stage. As a result, plant productivity did not exceed the ecosystem respiration.

## Discussion

### Burn severity uncertainties

The uncertainties caused by using dNBR to estimate the burn proportion can be addressed in five aspects. First, the reliability of using dNBR to estimate CBI varies. Although relatively high correlations between dNBR and CBI are found in many black spruce (*Picea mariana*)-dominated boreal forests^36–39^, CBI performs poorly in estimating the proportion of canopy combustion^40^ (R^2^ = 0.15). Furthermore, the correlation between dNBR and overstory CBI is relatively poor^38^ (R^2^ = 0.31–0.37), while the correlation between overstory CBI and the proportion of vegetation combustion is better^40^ (R^2^ = 0.44). This indicates that the dNBR has a significant uncertainty in estimating the proportion of canopy combustion. Overstory CBI is reported to saturate at high values and hardly increase as the dNBR value increases^38^. Therefore, the proportion of vegetation combustion could be underestimated for severe fires.

Second, the studies using dNBR to estimate combustion emission indicate that dNBR saturates when reaching approximately 1000 and hardly detects higher field burn severity^41^. This may also contribute to the lower combustion emission compared with those studies estimated by wood fuel types (e.g., black spruce, deciduous forest, and low shrub)^29,42^. However, the influence of the dNBR saturation should be limited since there are very rare fires with a mean dNBR value higher than 1000.

Third, environmental factors such as moisture condition, temperature, slope, elevation and time of burn were not considered in our study. However, studies have suggested that these factors could influence the burn severity^42,43^. In particular, fire area and emission tend to peak in summer in response to high vapor pressure deficit^44^, and the fuel tend to be wetter and more difficult to burn at lower elevation sites^45^. Therefore, including environmental factors and time of burn may improve the correlation between the dNBR and ground combustion proportion.

Fourth, the relationship between the dNBR and combustion proportion has been established for black spruce-dominated forests. However, part of the NA boreal forests is dominated by white spruce (*Picea glauca*) or pines (e.g., *Pinus banksiana*). A previous study has found no difference on the dNBR-CBI relationship between black spruce and pine dominated boreal sites^46^. However, the black spruce forest shows higher dNBR values than the white spruce forest under a given field burn severity index due to greater canopy combustion^41^. When using the dNBR-CBI relationship derived from black spruce forest to estimate the CBI of a white spruce forest fire, the CBI value and the burn emission would be underestimated.

Finally, although soil combustion is an important source of boreal fire emission, dNBR has uncertainties in estimating soil burn severity. For example, dNBR detects soil combustion partly because fire changes soil hydraulic conditions, while the relationship between soil hydraulic conditions and dNBR is also influenced by soil texture^47^, bulk density and soil organic and gravel fraction^48^.

In addition to the uncertainties caused by utilizing dNBR, without classifying boreal forests into more detailed ecozones could also cause uncertainties. Previous studies suggested that the fractions of different types of fuel vary among ecozones, and their fuels respond differently to burn severity^29,49^. In addition, the relationship between CBI and soil/vegetation C and N combustion is derived based ona limited number of black spruce samples, which might not adequately represent other forest types^40^. Since the relationships between dNBR and the combustion completeness of each type of fuel or between CBI and soil/vegetation combustion fraction of each ecozone are not available, this study made a compromise to use the data of black spruce-dominated forest to represent various boreal forest types. We recognized this will induce uncertainty in our analysis.

### Combustion emissions

The uncertainties of using dNBR to estimate regional fire combustion come from various sources, which are difficult to quantify. However, the advantage of using dNBR is that it uniquely describes the burn severity of each fire event, which at least in part constrain the overall uncertainty. In order to examine the effectiveness of using dNBR in model simulations, we compare fire direct emission estimated by our study to previous studies.

The combustion emission is influenced by C stock at the time of fire. Our simulations suggest 10.1 kg C m^−2^ in the soil pool, which is similar to the previous estimates^16,32,50,51^. The vegetation C pool is 2.2 kg C m^−2^, which is lower than values reported by other studies by around 1.0 kg C m^−2^^50–52^. At the regional scale, our vegetation C stock in NA boreal area is 14.0 Pg C, while literature suggests 8.9–14.0 Pg C^50,52,53^; our soil C stock is 60.6 Pg C, which also agrees with the report of 53.2–66.7 Pg C^50^. Given this reasonable estimation of C pool, our estimated C emissions per unit area during the fire were lower than that in some previous studies in both Alaska and Canada. However, it falls within the range of some of previous studies (Supplementary Table S3). The possible reason for our estimation being lower than some field measurements is that field measurements tend to do sampling in core burn areas in the fire perimeter more than from unburned and low-severity patches. However, these patches are included in the fire perimeter used to extract the dNBR values and result in a lower mean severity in our study. According to an Alaska field study^41^, the mean combustion emission within the fire perimeter (1.98 ± 0.34 kg C m^−2^) was lower than the mean in the core burn area (2.67 ± 0.40 kg C m^−2^) and at the field sites (2.88 ± 0.23 kg C m^−2^). A study in the southern Canadian fire in 2015 comparing modeled and measured combustion emission also suggested that the model estimation is lower than the measurement by 0.8 kg C m^−2^. This difference exists partly because the regional average carbon stock per unit area is lower than that at the field sites^54^.

During 1986–2016, the average regional combustion emission was 7.2 Tg C year^−1^ for Alaska, higher than the 50-year average^55^ (Table 1). Compared with the previous estimation^45^, our estimation for emissions during 2001–2012 is lower, which is expected since our emission rate per unit area is also lower (Supplementary Table S3). Meanwhile, our estimation for 2004 is slightly lower while for 2006–2008 it is higher^42^. Moreover, all of our estimations for the boreal area are lower than the combustion emissions for the entire Alaska during the same period^56–58^. These are reasonable differences, since about 87% of the fire events occurred in forests and taiga woodlands in Alaska^56^. For Canada, the average combustion emission was 49.9 Tg C year^−1^. Our estimation of annual average during 1990–1999 is higher than previously reported values^29,57^, as a result of higher emission rate per unit area. However, the value for 1990–2008 falls within the range reported by previous studies^49,59^. For North American boreal forests, our estimation was 57.1 Tg C year^−1^ for 1986–2016 and 44.9 for 1997–2009, which is lower than the previous study with the lower emission rate per unit area^60^. However, the average emission during 1997–2016 (50.7 Tg C year^−1^) is very close to the estimation by a previous study^61^ (51.0 Tg C year^−1^).

**Table 1:** Comparison on regional combustion emission per year (*shows the estimation for entire Alaska/Canada).

Region	Time	Combustion (Tg C year^−1^)	Source
Alaska	1986–2016	7.2	This study
	1950–2000	5.9	French et al.^55^
	1940–2012	10.7 ± 4.0*	Chen et al.^56^
	1990–2012	18.2 ± 2.7*	Chen et al.^56^
		7.6	This study
	1950–2009	12.5	Genet et al.^85^
		7.0 ± 1.0	Turetsky et al.^86^
	2001–2012	15.0	Veraverbeke et al.^45^
		10.0	This study
	2004	42.4	Kasischke and Hoy^42^
		40.5	This study
	2006–2008	0.6	Kasischke and Hoy^42^
		1.2	This study
	1990–1999	9.4*	Goetz et al.^57^
		4.7	This study
	2000–2005	27.8*	Goetz et al.^57^
		16.1	This study
	2002–2006	21.8*	Wiedinmyer and Neff^58^
		18.0	This study
Canada	1986–2016	49.9	This study
	1959–1999	27.0 ± 6.0	Amiro et al.^29^
	1990–1999	39.0	Amiro et al.^29^
		42.2*	Goetz et al.^57^
		46.4	This study
	1940–2012	47.8 ± 7.4*	Chen et al.^56^
	1990–2012	57.9 ± 8.7*	Chen et al.^56^
		38.4	This study
	1990–2008	27.0 ± 19.0	Stinson et al.^49^
		24.0 ± 19.0	Kurz et al.^59^
		38.1	This study
North America	1986–2016	57.1	This study
	1997–2009	54.0	van der Werf et al.^60^
		44.9	This study
	1997–2016	51.0	van der Werf et al.^61^
		50.7	This study

### Post-fire C dynamics

The differences in C balance between pre-fire and post-fire conditions are mainly in two aspects: net plant productivity (NPP) and soil respiration (R_H_). In our simulation, NPP increases linearly after the fire, which is consistent with studies using process-based model and/or satellite data to estimate NA boreal forest post-fire NPP recovery^62–64^. We estimate that fires cause NPP reduction by 170.5 g C m^−2^ year^−1^ on average, which agrees with the range estimated by satellite NDVI for NA boreal forest^62,63^ (60–260 g C m^−2^ year^−1^ and 126.8–216.7 g C m^−2^ year^−1^). The trend of post-fire NPP is in close agreement with the simulated net N mineralization rate (Fig. 4b,c). During the year of fire, net N mineralization rate decreases likely due to the massive reduction in soil N^34^. In agreement with this study, both previous TEM simulation^34^ and field measurements^65^ show the same trend of decrease in net N mineralization immediately after the fire and then a gradually increases to the pre-fire condition. With the recovery of net N mineralization, more N becomes available to plants, triggering a faster recovery. In addition to net N mineralization, the time NPP takes to recover is also influenced by burn severity (Fig. 4c). Even light fires require more than 25 years to recover. However, the dataset from Boreal Plains ecozone of Alberta showed NPP becomes stable in 20–30 years after fire^64^, and satellite estimation reports an even shorter NPP recovery time (within 10 years after fire)^62^. The results from previous studies are consistent with our results^66,67^, NPP peaks when the stand age is 50–75 years. Furthermore, an even longer recovery time has been previously suggested with NPP peaks in 80–100 years after the fire^34^.

R_H_ is influenced by soil moisture, soil temperature, soil organic C content and microbial community^24^. Although microbial community shift under fire disturbance is not considered by the model, the change in soil temperature, soil moisture and soil organic C could partly explain the change in R_H_. Our results show that soil moisture increases after fire, with a higher increase in more severe fires. This is consistent with the previous findings^20^, suggesting that such a behavior is attributed to a decline in vegetation water uptake and soil infiltration rates^68^. Soil temperature increases after fire, in agreement with field observation^17,20^, and a model simulation^34^. The magnitude of the change reported here (1–2 °C) is close to the values reported by a previous modelling study (e.g., 1.5–4.5 °C^34^). However, a previous field measurement suggests higher values (5–8 °C^17^), the reason of which could be that their measurement is in non-permafrost area, while our result is generated from both permafrost and non-permafrost areas. In addition to increasing soil temperature and moisture, fire also increases the temperature sensitivity of microbial respiration (i.e., Q_10_^69^). In our simulation, when the fire was relatively less severe, i.e., dNBR < 300, the soil microbial activities are more intense under moister and warmer conditions. However, since the soil is hardly burned, the negative effect of soil organic C decline is minor and could not overwhelm the positive effect of wetter and warmer soil condition on R_H_. This agrees with a previous report^17^. On the contrary, when dNBR is higher than 300, the negative effect of soil organic C decrease would offset the increased microbial activity, resulting in a lower R_H_ (Fig. 4g).

However, in our simulation, R_H_ decreased likely due to the lower microbial abundance^70^ and the decreased soil organic C when the dNBR is higher than 300 (Fig. 4g). This trend is consistent with field measurement^13^ and model estimation^34^. Similarly, R_H_ decreases shortly after fire in a Canadian boreal forest site^71^, and a study on the entire boreal area suggests that around three decades for R_H_ to stabilize after fire^23^. On the contrary, when burn severity is low and the soil is not combusted, decline in R_H_ was not observed in our study. Regardless of the burn severity, the post-fire R_H_ tends to account for a certain proportion of the total fire-related emissions (Supplementary Fig. S2c,d). This post-fire emission is reported to be almost three times as large as the direct emission in the Northern Hemisphere as reported in a previous modelling study^72^.

In our simulation, NEP recovered almost to the pre-fire level in the 25th year after fire (Fig. 4h), while a previous modelling study indicates forest does not become a C sink until 35–50 years after fire^34^. This difference might result from the different burn severities used in our simulations. In addition, whether a forest becomes a C sink or a source after fire in a given period also differs by the species composition and climate at the site^3^. However, it should be noted that even if the NEP of a forest ecosystem is positive, it is not necessarily a ‘true C sink’ as long as the C emitted during combustion is not compensated by the post-fire plant productivity. In our simulation, even if the cumulative NEP is positive (59 Tg C), the NA boreal ecosystem is still a net C source since net C assimilation did not exceed combustion emissions. As a result, the C storage in both soil and vegetation keeps decreasing (Supplementary Fig. S1b,c). This is supported by the finding that Canadian boreal ecosystems had become a C source in the 1980s, when other disturbance factors such as insects, clear-cur harvesting were considered^2^. A more recent model simulation by TEM also suggested that the NA boreal forest is a net source of 27 Tg C year^−1^ during 1987–2016, and 52 Tg C year^−1^ during 1997–2006^73^, which agrees with our result. Similarly, the northern high latitudes (above 50° N) are reported to be a current C source by 276 Tg C year^−1^, although more ecosystem types other than boreal forests are included^74^.

It should be noted that our model still oversimplifies the fire impacts on the complex ecosystem processes. For example, while the fire-induced soil temperature increase and active layer deepening^75^ is modeled, the changes of soil hydrological properties following permafrost thaw is not considered in TEM. Similarly, the effects of thermokarst-induced land morphology changes on C dynamics are not considered. If the impact of permafrost thaw on soil hydrological properties was considered, the post-fire soil moisture could be higher and R_H_ could be different. Furthermore, the water released from permafrost thaw could also change the drainage pattern, thereby affecting ecosystem structure^76^. Satellite images show that some boreal forests are more dominated by deciduous species during post-fire succession^77^, but this change is not considered in the present simulation. Because the productivity of deciduous and coniferous forests is different, considering vegetation dynamics shall help constrain our future quantification uncertainty. In addition, the less flammable and more reflective deciduous-dominated forests could reduce the impact of future climate change on fire occurrence^78^. Therefore, better knowledge on the landscape changes shall help improve the accuracy of our C estimates.

## Methods

### Overview

We extracted the dNBR value for 23,750 NA boreal fires during 1986–2016 via Google Earth Engine (GEE) to represent the burn severity. dNBR values were further correlated with Composite Burn Index (CBI), a field-measured burn severity index, which was used to estimate the proportion of vegetation and soil C consumption in the Terrestrial Ecosystem Model^34^. Model inputs include monthly air temperature, precipitation, vapor pressure and cloudiness, soil texture, plant functional type, elevation and annual CO_2_ concentration (Mauna Loa). Three fire areas in Canadian boreal forest with observation data were used to evaluate the model. Regional simulations were conducted for Alaskan and Canadian boreal forests to quantify the C budget under fire impact during 1986–2016. Notably, the other important disturbances such as insects, harvest, land use and land cover change are not considered in this study.

### Burn severity estimation

The fire history data for Alaska and Canada are available in Alaska Interagency Coordination Center and Natural Resources Canada, respectively. These records were spatially intersected with the boundary of North American boreal forest provided by Natural Resources Canada so that only boreal forest fires were kept. The fire year, fire perimeter and fire area were recorded, while the burn severity data was not available (Fig. 1).

Since the 1980s, the estimation of burn severity with satellite data became possible. Current fire-related satellite indices include difference Normalized Burn Ratio (dNBR) and relative differenced Normalized Burn Ratio (RdNBR). Both dNBR and RdNBR are calculated from Normalized Burn Ratio (NBR), which are defined by the near infrared (Band 4) and short-wave infrared (Band 7) bands of Landsat TM/ETM data^79^:

1 $$NBR=\frac{\left(B4 - B7\right)}{(B4 + B7)} \times 1000,$$

2 $$dNBR={NBR}_{prefire}- {NBR}_{postfire.}$$

RdNBR is simply the relative form of dNBR and both of their values positively correlate with burn severity. In particular, a dNBR value below 100 tends to indicate no-fire, while the dNBR value for burned area usually ranges between 100 and 1300, with the average of 200–400 reported in Alaska boreal field sites^36,80^. Although RdNBR performs better than dNBR for burn severity classification, the correlations between RdNBR and dNBR with field burn severity indices are very close^39^. We thus extracted the mean dNBR value for each fire event in the North American boreal forest area during 1986–2016 via Google Earth Engine (GEE) to represent burn severity. The ${NBR}_{prefire}$ is the NBR value of the fire area in the year before fire, while the ${NBR}_{postfire}$ is the NBR of the same area in the year after fire (Eq. (2)). Only images taken during summer (Jul. 15th–Sep. 15th) are used to calculate NBR so that the fire impact on the forest ground could be maximized. For each fire, its mean dNBR value was subtracted by a background dNBR to remove the background variation. The background value was initially defined as the mean dNBR value in a buffer zone at the year of fire, while the buffer zone was the area between 1500 and 1800 m out of the fire boundary. In case of creating a buffer-zone takes up a large GEE’s computation capacity, the dNBR value during 1 year before fire within the fire perimeter was used as background value instead. Although these two methods show some deviations at the low-value end, they generally fall on the 1:1 line (Supplementary Fig. S3a). Among the total of 23,750 (Alaska: 2346 versus Canada: 21404), 126 (Alaska: 51 versus Canada: 75) fire events do not have available images due to the limitation of satellite coverage. Their dNBR values were estimated from the average of ten fires closest in size (Fig. 2b showing the gap-filled dNBR of all North American boreal fires in 1986–2016).

Although there is no study to directly relate dNBR to the proportion of C removal during a fire, it is possible to build up their indirect relations. Many studies have proposed or reviewed the correlations between dNBR and a field-based burn severity index, the Composite Burn Index (CBI), in boreal forests^36,37,39^. When measuring CBI, forests are divided into five layers vertically, and a CBI score is given to each layer according to the post-fire condition. Then these five scores are combined into a total CBI along a 0–3 scale, with higher values representing more severe burning^80^. The correlation between CBI and dNBR in our study was based on published field data^39,81^ in Canadian boreal forests (Supplementary Fig. S3b). The linear regression equation is:

3 $$CBI=0.0023 \times dNBR+0.5561 \left({R}^{2}= 0.57\right).$$

Therefore, for each fire event, the CBI value was estimated from its dNBR value. Based on the field measurements of 38 black spruce (*Picea mariana*) dominated boreal forest sites, a previous study has established a linear relationship between CBI and the proportion of C removal in vegetation and soil^40^:

4 $$Organic\, soil \,C \,combustion \left(\%\right)=51.42 \times CBI-63.49 \left({R}^{2}=0.50\right),$$

5 $$Canopy\, C\, combustion \left(\%\right)=14.15 \times CBI+48.63 \left({R}^{2}=0.15\right).$$

These equations were used to estimate soil and vegetation C removal based on CBI values. Notably, the correlation between CBI and the proportion of vegetation C combustion is relatively low, which also introduces uncertainties to C emission modelling. In addition, this relationship between dNBR and combustion proportion is based on black spruce dominated boreal forests. This influence should be acceptable since the majority of C is stored in soils rather than vegetation.

### Model and data

TEM is a process-based biogeochemical model that simulates C and nitrogen (N) dynamics at regional scales. The model has been used previously to simulate fire impacts on C dynamics of black spruce-dominated boreal forests in Alaska^34^. In this version, TEM is integrated with a hydrology module and a soil thermal module. After fire disturbance, foliage is assumed to be linearly recovering for the first 5 years, and then tends to show a sigmoid trend. Moss layer thickness recovery is described by an exponential function of the year after fire. Simulated net N mineralization dynamics shows a close agreement with the trend of vegetation C. The model captures field measurements well at a fire chronosequence in Alaska. A more detailed description of the model structure and parameters can be found in supplementary information and ref^34^. Here we use the model to simulate the fire impacts on C dynamics of North America boreal forests. The model was first updated from a serial version into a parallel version to efficiently conduct large-scale simulations. After that, dNBR was incorporated into model simulations as an input variable to account for the impacts of burn severity (Eqs. (3)–(5)).

Monthly air temperature (°C), vapor pressure (hPa), precipitation (mm) and cloud cover (percentage) data were used to drive the model. The climate record (1901–2016) derived from observations and resampled into 0.5° × 0.5° grid was provided by the Climate Research Unit of the University of East Anglia (version 4.03)^82^. In addition, spatially-explicit data of soil texture (percentage of silt, clay and sand^83^), elevation^34^ and plant functional type^84^ were also used. Atmospheric CO_2_ data were obtained from Mauna Loa annual CO_2_ records provided by Global Monitoring Laboratory, Earth System Research Laboratories. Fire data including fire year and burn severity are discussed in Section of burn severity estimation.

### Model verification

The model was calibrated using the field data from black spruce forest ecosystems in interior Alaska in previous work, where the model agreed with field observations in terms of post-fire 10 cm soil temperature, 20 cm soil temperature, soil heterotrophic respiration (R_H_) and soil organic C^34^. The parameters in this study is adopted from the previous work^34^, and we test the applicability of these parameters at three Canadian boreal sites. The modeled and measured soil C and vegetation C agreed, while a small discrepancy on soil temperature and soil N is found (Supplementary Table S4). These sites were burned in 1969, 1990 and 2012, respectively, and are dominated by black spruce and white spruce. For these sites, vegetation C, soil organic C, soil N, 5 cm soil temperature and 10 cm soil temperature were measured in August 2015^69,71^.

Before carrying out simulation for these sites, their burn severity should be defined. Although extracting the dNBR for the fires in 1990 and 2012 was feasible, there was no satellite record for the fire in 1969. However, the proportion of soil combustion can be coarsely estimated from soil organic matter depth, which was observed for these sites^40^. For the fire in 1990 and 2012, the approximate soil combustion proportions were 40% (10.2 cm organic layer remaining) and 65% (5.0 cm organic layer remaining), respectively. The dNBR values calculated from the correlation between the proportion of soil C removal were 633.28 and 844.67, respectively. The actual dNBR values for 1990 and 2012 sites were then extracted from GEE for comparison. The calculated and actual dNBR values were close (633.28 versus 686.72, and 844.67 versus 811.49, Supplementary Table S4). Therefore, for the site burned in 1969, it is reasonable to estimate the input dNBR value from the depth of the soil organic layer, with 506.5 corresponding to an organic layer depth of 14.1 cm.

In terms of C stocks, the model estimated vegetation C and soil organic C tend to fall within the range of field measurement, except for the vegetation C at the site burned in 1990 (measurement: 698.9 ± 178.2 versus estimated: 889.3) (Supplementary Table S4). However, since the model estimation is only 12.2 g C m^−2^ higher than the upper bound of the field measurement, we assume the model is still reliable in estimating field C stocks. For soil temperature, the 5 cm soil temperature at the site burned in 1990 and the 10 cm temperature at the site burned in 1969 showed discrepancies between model estimation and field measurement. However, these discrepancies are not large. In particular, for the former, the estimation is 1.3 °C lower than the lower bound of measurement; while for the latter, the estimation is 0.9 °C higher than the upper bound of measurement. The soil organic N, model estimation tends to be higher or lower than the observation. However, the discrepancy between modeled and measured soil organic N is not large, which will not affect the estimation of C dynamics under the fire disturbance (Supplementary Table S4).

### Regional carbon dynamics simulations

Two regional simulations were conducted with and without considering the impacts of fire disturbance. In the no-fire simulation, the North American boreal forest was gridded into 0.5° × 0.5° cells and the proportion of forest area within each cell was calculated. After spinning up for 120 years, a transient simulation was conducted for each cell during 1986–2016. When considering fire impacts, the fire polygons were dissected into units with unique fire history. Each unit was intersected with the 0.5° × 0.5° grid to create ‘cohorts’ with unique cell coordinate and fire history^26^. Then the area proportion of each cohort out of the boreal forest in the same cell was calculated. We run the simulation for each cohort, and the output values of each cohort and the no-burn areas were weighted by their area to get the mean of the cell.

When analyzing the C stock and flux of the entire North American (NA) boreal forest region, for each cell, the mean value of soil organic C, vegetation C, net ecosystem productivity (NEP), net primary productivity (NPP) and R_H_ were multiplied by the area of boreal forest in that cell to get the cell total value. The aggregation of all cells is the total value for the NA boreal forests. During 1986–2016, at the regional scale, the C balance (CB) under no fire disturbance is calculated as the accumulative NEP.

By considering fire impacts, the regional carbon sink and source activities (C balance (fire), CBF) are the accumulative NEP minus accumulative fire consumption.

## Supplementary Information

Supplementary Information.

## Data Availability

All data used in this manuscript can be accessed in Purdue University Research Repository (https://purr.purdue.edu/publications/3532/1).
