# Supplementary material for: North American boreal forests are a large carbon source due to wildfires from 1986 to 2016
Source: Sci Rep. 2021 Apr 8;11:7723. doi: 10.1038/s41598-021-87343-3 (PMC8032736; doi:10.1038/s41598-021-87343-3)
Supplement: Supplementary file 1 — Supplementary Information. [file 41598_2021_87343_MOESM1_ESM.docx]

**Supplementary Information**

North American boreal forests are a large carbon source due to wildfires from 1986 to 2016

Bailu Zhao^1^, Qianlai Zhuang^1,2^, Narasinha Shurpali^3^, Kajar Köster^4^, Frank Berninger^5^, Jukka Pumpanen^6^

^1^Department of Earth, Atmospheric, and Planetary Sciences, Purdue University, West Lafayette, Indiana, 47907

^2^Department of Agronomy, Purdue University, West Lafayette, IN 47907

^3^Natural Resources Institute Finland (Luke)

^4^Department of Forest Sciences, University of Helsinki, PO Box 27, FI-00014 University of Helsinki, Finland

^5^Department of Environmental and Biological Sciences, University of Eastern Finland, PO Box 111, FI-80101 Joensuu, Finland

^6^Department of Environmental and Biological Sciences, University of Eastern Finland, PO Box 1627, FI-70211 Kuopio, Finland

* Correspondence to: [qzhuang@purdue.edu](mailto:qzhuang@purdue.edu)

The supplemental method of TEM simulation on fire disturbance

The fire history data used in this study provides fire boundary and fire year information, but not burn severity. In addition, the fire start and end time and fire duration are not always available. In TEM, we simply assume the fire removes certain fraction of vegetation and soil C and N in the last month of the fire year. This is achieved with two steps below in TEM:

1. The fractions of C and N removal are estimated solely based on dNBR, while the climate factors, fire occurrence time and duration are not used for burn severity estimation.
2. In TEM, for boreal forest, the litter fall is a static fraction of vegetation C and N every month. Although this might oversimplify the process of vegetation and soil C and N exchange, it makes the vegetation and soil C and N pools stable throughout the year. Therefore, removing certain fraction of C and N from these pools in December does not cause much difference from removing in another month.

In TEM, in addition to the removal of C and N pools, fires also impact the ecosystem in two major aspects ^1^.

1. Fires destroy moss. Moss layer is the uppermost layer in soil thermal module and its thickness impacts soil temperature. In the year of fire, TEM assumes the moss layer is completely combusted. After fire, moss thickness is described by an empirical function depends on the number of year after fire:

$$\mathrm{moss}_{\mathrm{thick}}=a\times(1-e^{\left( -b\times t \right)})$$

Where $\mathrm{moss}_{\mathrm{thick}}$ is the moss thickness in cm, a and b are parameters, and t is the year after fire.

1. Vegetation need time to recover after fire. One direct impact of vegetation combustion is GPP decrease. GPP in TEM is a function of canopy biomass development (FOLIAGE), which is on 0-1 scale. When there is no fire, FOLIAGE is a function of vegetation C. However, in the first five years after fire, FOLIAGE is assumed to be 0.05. In the sixth year after fire, FOLIAGE is again a function of vegetation C.

$$FOLIAGE= \frac{1}{1+m_{1}\times e^{m_{2}\times\sqrt{f(C_{V})}}}$$

Where$m_{1}$ and $m_{2}$ are parameters, $C_{V}$ is vegetation C.


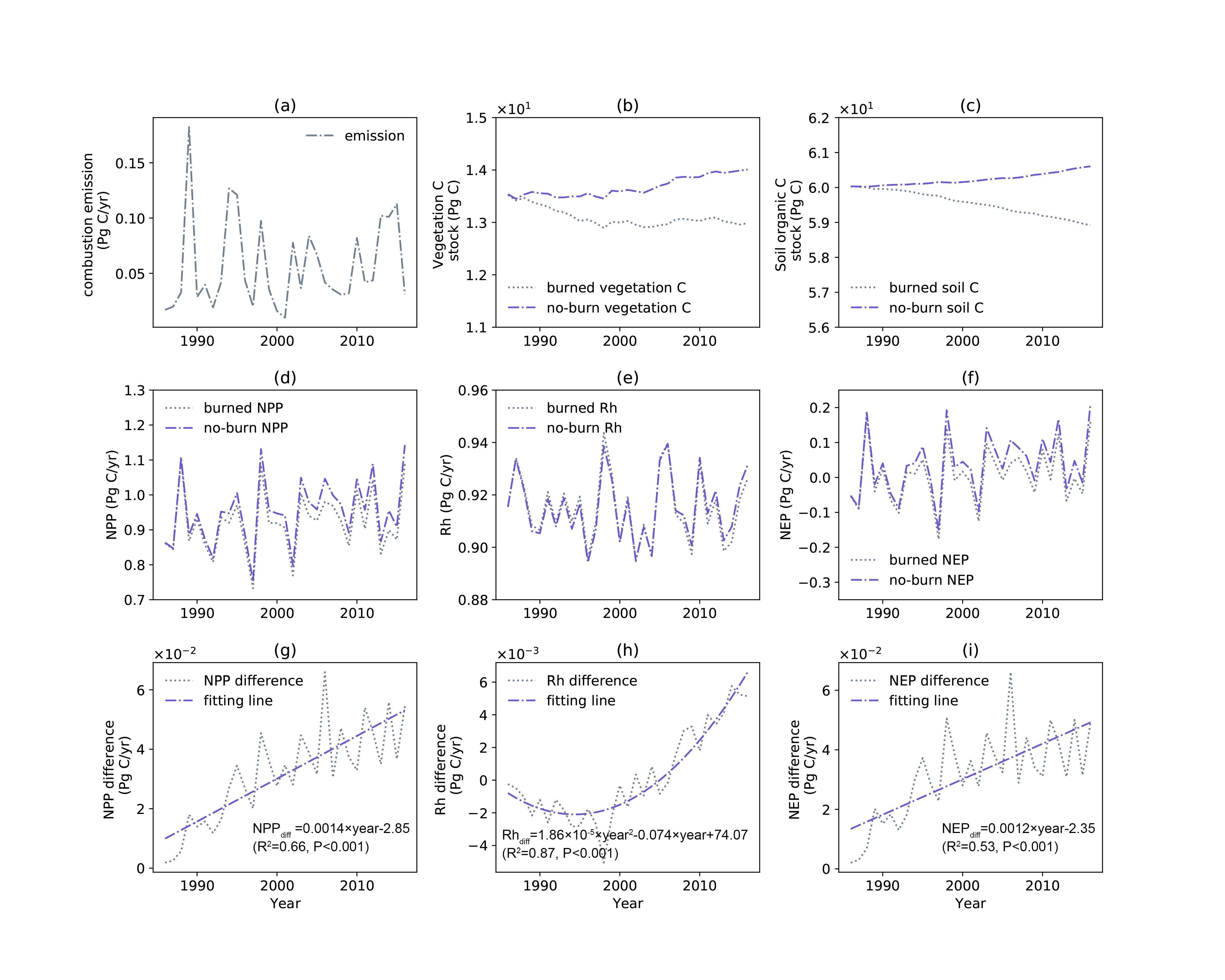


**Supplementary Figure 1.** Temporal variations (1986-2016) of ecosystem C flux and stock in NA boreal forests (flux in Pg C yr^-1^, stock in Pg C). The variation of (a) annual C emitted by biomass combustion; (b) annual regional average vegetation C storage under fire and no-fire scenario; (c) annual regional average soil organic C storage under fire and no-fire scenario; (d) annual regional total NPP under fire and no-fire scenario; (e) annual regional total R_H_ under fire and no-fire scenario; (f) annual regional total NEP under fire and no-fire scenario; (g) annual regional total NPP difference between fire and no-fire scenario and its fitting line; (h) annual regional total R_H_ difference between fire and no-fire scenario and its fitting line; (i) annual regional total NEP difference between fire and no-fire scenario and its fitting line.


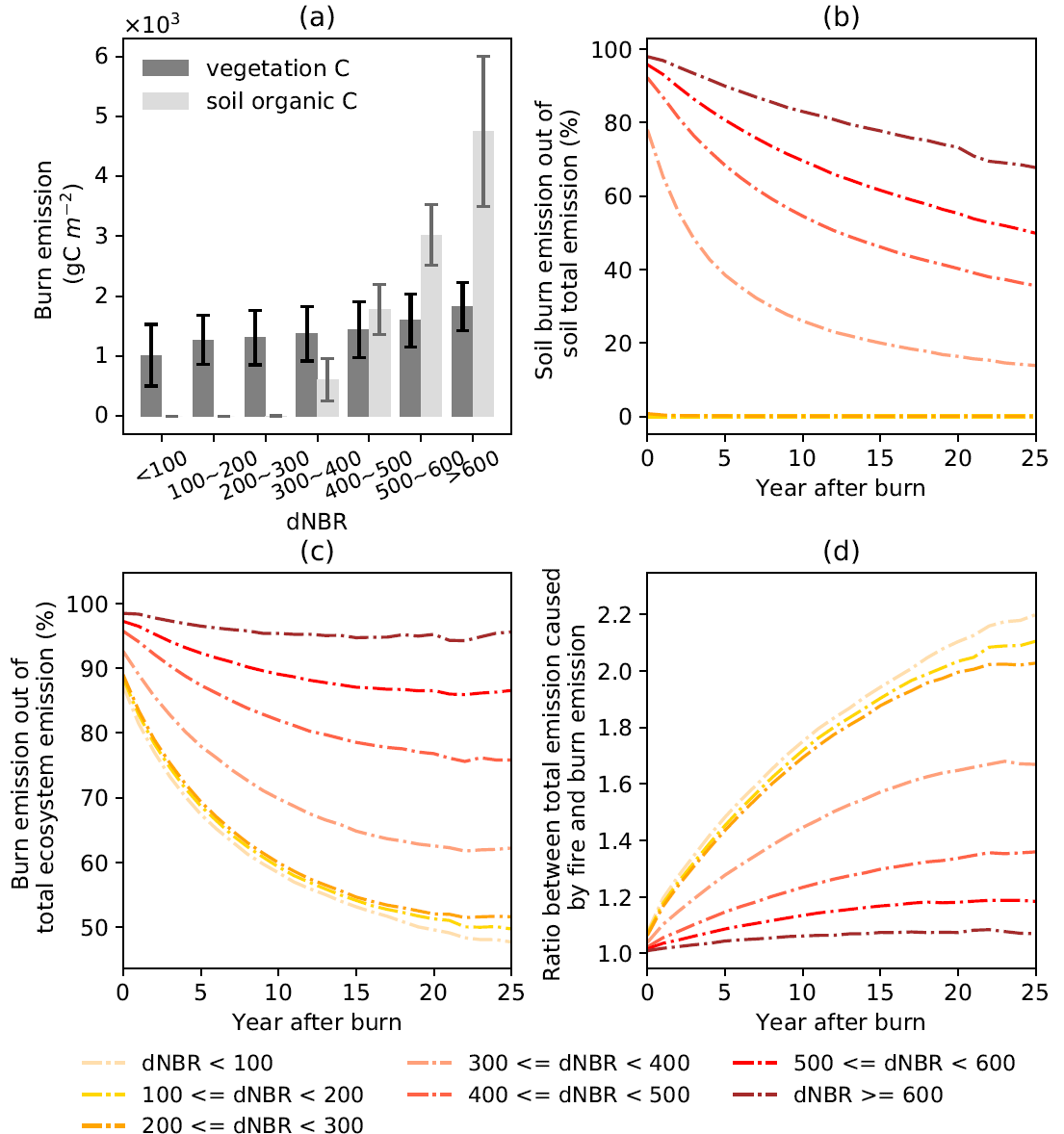


**Supplementary Figure 2.** Emission pattern during- and post-fires: (a) The amount of C combustion from vegetation and soils. The values are based on the average and standard deviation of all fire events. (b) In the total soil emission since the year of fire, the percentage contributed by soil organic combustion. (c) In the total ecosystem emission since the year of fire, the percentage contributed by combustion (vegetation plus soil emissions). (d) The ratio between the total emission related to fire (i.e., the emission difference between the fire and the no-fire scenario) and the during-fire emission. Figure (b), (c) and (d) are generated from simulations that burned only once.


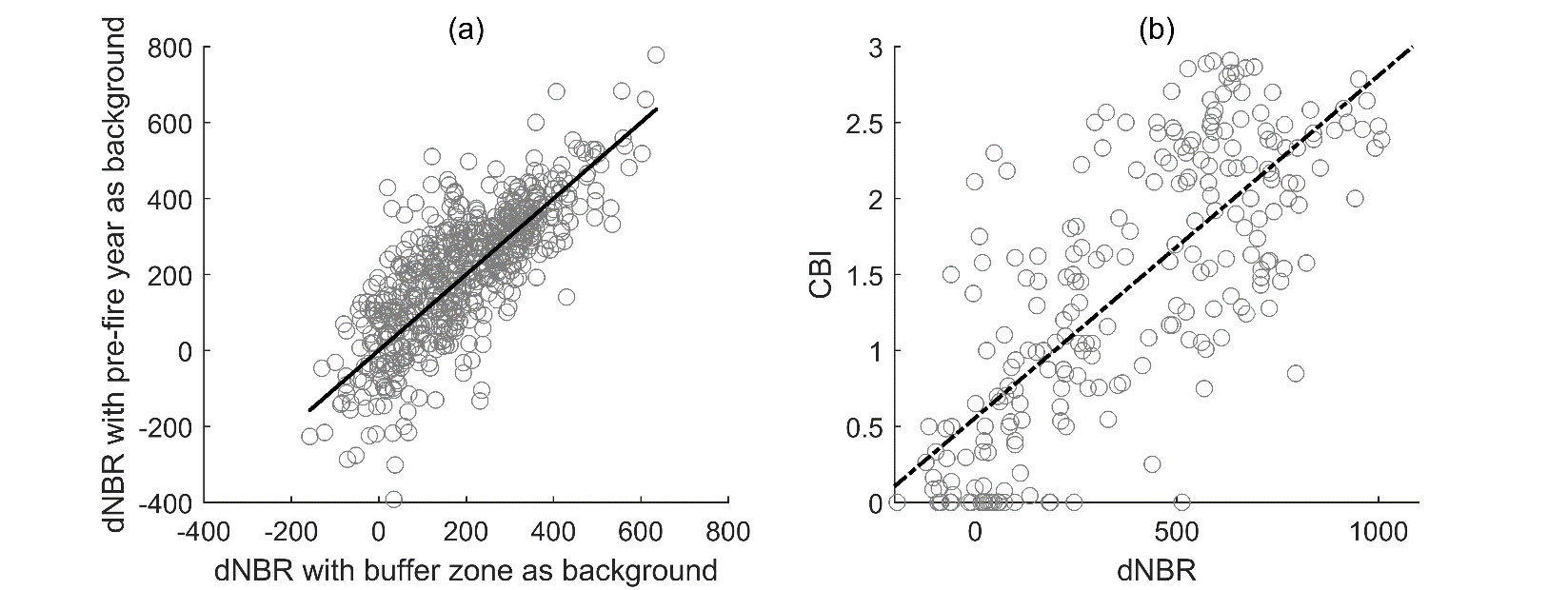


**Supplementary Figure 3.** Comparison between two methods of extracting dNBR and correlation between dNBR and CBI: (a) The comparison between two different methods to calculate the background dNBR. For the x axis, ‘buffer zone’ refers to the area between 1500 and 1800m out of the fire boundary. The black line represents the 1:1 line of x axis. The samples are all fire events in Alaska boreal forest during 2014-2018 (n = 634). (b) The linear correlation between dNBR and CBI (n = 234).

**Supplementary Table 1.** Area percentage of burn frequencies in NA boreal region (1986-2016)

| Burn frequency | Burn area(km^2^) | Percentage |
| --- | --- | --- |
| 1 | 808112.5 | 95.18% |
| 2 | 39749.1 | 4.682% |
| 3 | 1004.3 | 0.118% |
| 4 | 49.2 | 0.006% |
| 5 | 35.1 | 0.004% |
| >5 | 109.7 | 0.013% |

**Supplementary Table 2.** Annual direct emissions of NA boreal fires (1986-2016)

| Year | Vegetation direct emission (Tg C) | soil direct emission (Tg C) | Total direct emission (Tg C) |
| --- | --- | --- | --- |
| 1986 | 15.78 | 1.20 | 16.98 |
| 1987 | 16.29 | 3.52 | 19.81 |
| 1988 | 30.42 | 2.28 | 32.70 |
| 1989 | 128.85 | 53.41 | 182.26 |
| 1990 | 27.62 | 0.97 | 28.59 |
| 1991 | 32.62 | 7.06 | 39.67 |
| 1992 | 14.59 | 4.54 | 19.13 |
| 1993 | 33.52 | 8.39 | 41.91 |
| 1994 | 90.97 | 35.88 | 126.85 |
| 1995 | 103.63 | 17.32 | 120.95 |
| 1996 | 30.63 | 12.95 | 43.58 |
| 1997 | 17.60 | 2.52 | 20.12 |
| 1998 | 64.96 | 32.40 | 97.36 |
| 1999 | 29.03 | 6.48 | 35.51 |
| 2000 | 11.71 | 4.19 | 15.90 |
| 2001 | 9.00 | 0.86 | 9.86 |
| 2002 | 55.57 | 22.11 | 77.68 |
| 2003 | 29.72 | 6.90 | 36.62 |
| 2004 | 71.48 | 12.32 | 83.80 |
| 2005 | 47.79 | 19.05 | 66.84 |
| 2006 | 28.76 | 12.92 | 41.68 |
| 2007 | 25.37 | 9.89 | 35.26 |
| 2008 | 25.83 | 4.86 | 30.69 |
| 2009 | 23.89 | 7.85 | 31.74 |
| 2010 | 51.16 | 30.84 | 82.00 |
| 2011 | 37.05 | 4.92 | 41.97 |
| 2012 | 30.60 | 12.99 | 43.59 |
| 2013 | 71.75 | 30.35 | 102.10 |
| 2014 | 65.28 | 35.82 | 101.10 |
| 2015 | 80.26 | 32.32 | 112.58 |
| 2016 | 23.01 | 7.96 | 30.97 |
| Total | 1324.74 | 445.07 | 1769.81 |

Supplementary Table 3. Comparison on combustion emission per unit area

| Region | Combustion (kg C m^-2^) | Method | Source |
| --- | --- | --- | --- |
| Alaska | **1.4** | **Estimated from dNBR and C stock** | **This study** |
|  | 2.5 | Use nonlinear multiplicative model to environmental variables, fire time, pre-fire tree cover and dNBR | Veraverbeke, et al. ^1^ |
|  | 2.0 ± 0.3 | Field measurement | Rogers, et al. ^2^ |
|  | 3.3 (1.5-4.6) | Field measurement | Boby, et al. ^3^ |
|  | 1.6 ± 0.6 | Field measurement | Randerson, et al. ^4^ |
|  | 1.7-3.0 | Estimated by fuel type | Kasischke and Hoy ^5^ |
|  | 3.1 ± 0.7 | Estimated from land cover, drainage type and C stock | Tan, et al. ^6^ |
|  | 3.0±0.12 | Field measurement | Turetsky, et al. ^7^ |
|  | 2.5 | Summary from previous work | Turetsky, et al. ^7^ |
|  | 2.2* | Estimated based on literature | Turquety, et al. ^8^ |
|  | 2.8* | Field measurement | Turquety, et al. ^8^ |
|  | 2.0 (1.7-2.4) | Estimated by severity, biomass and fire area | French, et al. ^9^ |
|  |  |  |  |
| Canada | **2.1** | **Estimated from dNBR and C stock** | **This study** |
|  | 1.3 (1.8-3.9) | Estimated by fuel type and ecozone | Amiro, et al. ^10^ |
|  | 3.2±0.5 | Estimated by Fire Behavior Prediction System | de Groot, et al. ^11^ |
|  | 4.3±1.1 | Estimated based on Boreal Fire Effects Model | de Groot, et al. ^11^ |
|  | 3.35 | Field measurement | Walker, et al. ^12^ |
|  | 3.3 ± 1.1 | Field measurement | Dieleman, et al. ^13^ |
|  | 2.5 ± 1.1 | Spatial modeling | Dieleman, et al. ^13^ |
|  | 1.9* | Estimated based on literature | Turquety, et al. ^8^ |
|  | 1.4-2.8* | Field measurement | Stocks, et al. ^14^ |
|  |  |  |  |
| North America | **2.0** | Estimated by fuel type | **This study** |
|  | 2.7 | Estimated based on tree cover, climate and fire persistence | van der Werf, et al. ^15^ |
|  |  |  |  |
| Global | 2.6-3.3 | Model simulation based on baseline conditions | Kasischke, et al. ^16^ |
|  | 1.1-2.8 | Estimated from Boreal Wildland-Fire Emissions Model | Kasischke, et al. ^17^ |

* The values in the original literature are in dry matter per unit area, which are multiplied by 0.5 to convert into C per unit area.

**Supplementary Table 4.** Comparison between modeled and field-measured variables

| Burn year | Soil organic layer thickness (cm) | Estimated soil combustion (%) | dNBR |  | | Vegetation C (g C m^-2^) | | Soil organic C (g C m^-2^) | | |
| --- | --- | --- | --- | --- | --- | --- | --- | --- | --- | --- |
|  |  |  | estimated | actual | | measured | modeled | measured | | modeled |
| 1969 | 14.1 | 25.0% | 506.5 | - | | 2148.7 ± 581.4 | 1718.7 | 5556.0 ± 3245.6 | | 5674.9 |
| 1990 | 10.2 | 40.0% | 633.3 | 686.7 | | 698.9 ± 178.2 | 889.3 | 7460.9 ± 8298.7 | | 5043.7 |
| 2012 | 5.0 | 65.0% | 844.7 | 811.5 | | 220.4 ± 88.4 | 186.7 | 2612.3 ± 1422.2 | | 3596.3 |
|  |  |  |  |  | |  |  |  | |  |
|  | Soil organic N (g N m^-2^) | | 5cm soil temperature (°C) | | | | 10cm soil temperature (°C) | | | |
|  | measured | modeled | measured | | Modeled | | measured | | modeled | |
| 1969 | 197.0 ± 4.1 | 258.3 | 7.6 ± 2.0 | | 9.2 | | 5.3 ± 1.1 | | 7.5 | |
| 1990 | 264.6 ± 10.4 | 209.9 | 9.8 ± 0.5 | | 8.0 | | 7.7 ± 0.9 | | 6.9 | |
| 2012 | 108.1 ± 2.4 | 124.4 | 9.6 ± 2.8 | | 7.8 | | 6.8 ± 2.1 | | 7.6 | |

**SI References**

1 Zhuang, Q. *et al.* Carbon cycling in extratropical terrestrial ecosystems of the Northern Hemisphere during the 20th century: a modeling analysis of the influences of soil thermal dynamics. *Tellus B* **55**, 751-776, doi:10.1034/j.1600-0889.2003.00060.x (2003).

2 Veraverbeke, S., Rogers, B. M. & Randerson, J. T. Daily burned area and carbon emissions from boreal fires in Alaska. *Biogeosciences* **12**, 3579-3601, doi:10.5194/bg-12-3579-2015 (2015).

3 Rogers, B. M. *et al.* Quantifying fire-wide carbon emissions in interior Alaska using field measurements and Landsat imagery. *Journal of Geophysical Research: Biogeosciences* **119**, 1608-1629, doi:10.1002/2014jg002657 (2014).

4 Boby, L. A., Schuur, E. A. G., Mack, M. C., Verbyla, D. & Johnstone, J. F. Quantifying fire severity, carbon, and nitrogen emissions in Alaska's boreal forest. *Ecological Applications* **20**, 1633-1647, doi:10.1890/08-2295.1 (2010).

5 Randerson, J. T. *et al.* The Impact of Boreal Forest Fire on Climate Warming. *Science* **314**, 1130, doi:10.1126/science.1132075 (2006).

6 Kasischke, E. S. & Hoy, E. E. Controls on carbon consumption during Alaskan wildland fires. *Global Change Biology* **18**, 685-699, doi:10.1111/j.1365-2486.2011.02573.x (2012).

7 Tan, Z., Tieszen, L. L., Zhu, Z., Liu, S. & Howard, S. M. An estimate of carbon emissions from 2004 wildfires across Alaskan Yukon River Basin. *Carbon Balance and Management* **2**, 12, doi:10.1186/1750-0680-2-12 (2007).

8 Turetsky, M. R. *et al.* Recent acceleration of biomass burning and carbon losses in Alaskan forests and peatlands. *Nature Geoscience* **4**, 27-31, doi:10.1038/ngeo1027 (2011).

9 Turquety, S. *et al.* Inventory of boreal fire emissions for North America in 2004: Importance of peat burning and pyroconvective injection. *Journal of Geophysical Research: Atmospheres* **112**, doi:10.1029/2006JD007281 (2007).

10 French, N., Kasischke, E. & Williams, D. Variability in the emission of carbon-based trace gases from wildfire in the Alaskan boreal forest. *J. Geophys. Res* **107**, doi:10.1029/2001JD000480 (2002).

11 Amiro, B. D. *et al.* Direct carbon emissions from Canadian forest fires, 1959-1999. *Canadian Journal of Forest Research* **31**, 512-525, doi:10.1139/x00-197 (2001).

12 de Groot, W. *et al.* Estimating direct carbon emissions from Canadian wildland fires. *International Journal of Wildland Fire* **16**, doi:10.1071/WF06150 (2007).

13 Walker, X. J. *et al.* Cross-scale controls on carbon emissions from boreal forest megafires. *Global Change Biology* **24**, 4251-4265, doi:10.1111/gcb.14287 (2018).

14 Dieleman, C. M. *et al.* Wildfire combustion and carbon stocks in the southern Canadian boreal forest: Implications for a warming world. *Global Change Biology* **26**, 6062-6079, doi:10.1111/gcb.15158 (2020).

15 Stocks, B. *et al.* Crown fire behaviour in a northern jack pine - Black spruce forest. *Canadian Journal of Forest Research-revue Canadienne De Recherche Forestiere - CAN J FOREST RES* **36**, doi:10.1139/X06-185 (2004).

16 van der Werf, G. R. *et al.* Global fire emissions and the contribution of deforestation, savanna, forest, agricultural, and peat fires (1997–2009). *Atmos. Chem. Phys.* **10**, 11707-11735, doi:10.5194/acp-10-11707-2010 (2010).

17 Kasischke, E. S., Christensen, N. L. & Stocks, B. J. Fire, Global Warming, and the Carbon Balance of Boreal Forests. *Ecological Applications* **5**, 437-451, doi:10.2307/1942034 (1995).

18 Kasischke, E. S. *et al.* Influences of boreal fire emissions on Northern Hemisphere atmospheric carbon and carbon monoxide. *Global Biogeochemical Cycles* **19**, doi:10.1029/2004GB002300 (2005).
